# Reduced CD27^−^IgD^−^ B Cells in Blood and Raised CD27^−^IgD^−^ B Cells in Gut-Associated Lymphoid Tissue in Inflammatory Bowel Disease

**DOI:** 10.3389/fimmu.2019.00361

**Published:** 2019-03-05

**Authors:** Chathyan Pararasa, Na Zhang, Thomas J. Tull, Ming H. A. Chong, Jacqueline H. Y. Siu, William Guesdon, Konstantia Maria Chavele, Jeremy D. Sanderson, Louise Langmead, Klaartje Kok, Jo Spencer, Anna Vossenkamper

**Affiliations:** ^1^Peter Gorer Department of Immunobiology, King's College London, Guy's Hospital, London, United Kingdom; ^2^Obstetrics and Gynecology Hospital, Institutes of Biomedical Sciences (IBS), Fudan University, Shanghai, China; ^3^Blizard Institute, Barts and the London School of Medicine and Dentistry, Queen Mary University London, London, United Kingdom; ^4^Department of Surgery, Addenbrookes Hospital, University of Cambridge, Cambridge, United Kingdom; ^5^Department of Gastroenterology, Guy's and St Thomas' NHS Foundation Trust, London, United Kingdom; ^6^Department of Gastroenterology, Royal London Hospital, Barts Health, London, United Kingdom

**Keywords:** memory B cells, inflammatory bowel disease, GALT, mass cytometry, biologics, ustekinumab, infliximab

## Abstract

The intestinal mucosa in inflammatory bowel disease (IBD) contains increased frequencies of lymphocytes and a disproportionate increase in plasma cells secreting immunoglobulin (Ig)G relative to other isotypes compared to healthy controls. Despite consistent evidence of B lineage cells in the mucosa in IBD, little is known of B cell recruitment to the gut in IBD. Here we analyzed B cells in blood of patients with Crohn's disease (CD) and ulcerative colitis (UC) with a range of disease activities. We analyzed the frequencies of known B cell subsets in blood and observed a consistent reduction in the proportion of CD27^−^IgD^−^ B cells expressing all Ig isotypes in the blood in IBD (independent of severity of disease and treatment) compared to healthy controls. Successful treatment of patients with biologic therapies did not change the profile of B cell subsets in blood. By mass cytometry we demonstrated that CD27^−^IgD^−^ B cells were proportionately enriched in the gut-associated lymphoid tissue (GALT) in IBD. Since production of TNFα is a feature of IBD relevant to therapies, we sought to determine whether B cells in GALT or the CD27^−^IgD^−^ subset in particular could contribute to pathology by secretion of TNFα or IL-10. We found that donor matched GALT and blood B cells are capable of producing TNFα as well as IL-10, but we saw no evidence that CD27^−^IgD^−^ B cells from blood expressed more TNFα compared to other subsets. The reduced proportion of CD27^−^IgD^−^ B cells in blood and the increased proportion in the gut implies that CD27^−^IgD^−^ B cells are recruited from the blood to the gut in IBD. CD27^−^IgD^−^ B cells have been implicated in immune responses to intestinal bacteria and recruitment to GALT, and may contribute to the intestinal inflammatory milieu in IBD.

## Introduction

Inflammatory bowel disease (IBD) encompasses two clinical entities: Crohn's disease (CD) and ulcerative colitis (UC). Both are chronic debilitating diseases characterized by relapsing intestinal inflammation. While the exact etiology of both diseases is still not clearly understood, current literature suggests that an aberrant immune response to the intestinal flora contributes to pathology in genetically susceptible individuals (1–3). The evidence for this is however stronger for CD than for UC (4), which can be associated with autoimmune conditions like primary sclerosing cholangitis (PSC) in a small subgroup of patients (5). CD is a condition hallmarked by granulomatous transmural inflammation that can affect the entire gastrointestinal tract and is frequently complicated by fistulae and strictures. In contrast, UC is predominantly a disease affecting the rectum and distal colon, where it manifests as ulceration limited to the mucosal layer. However, rectum-sparing forms of UC are known to exist and in rare cases the pathology can extend into the terminal ileum (6, 7). Whilst big strides have been made in developing biologic therapies for both diseases, a significant proportion of patients with CD and UC remain treatment refractory, highlighting the unmet need for a better understanding of the mechanisms contributing to the pathogenesis of both diseases (8, 9).

Most immunological research in CD and UC has focused on T lymphocytes and macrophages that infiltrate the mucosa in active disease (10–12). Th1 and Th17 cells have been implicated especially in the pathogenesis of CD, whilst their role in UC is still not fully understood (13, 14). UC is also characterized by the infiltration of neutrophils into the inflamed mucosa, where they are proposed to have a pathogenic role by the maintenance of inflammation (15). However, in both CD and UC, a significant increase in the number of plasma cells in the lamina propria has been observed and linked to disease pathogenesis (16–18).

The gut-associated lymphoid tissue (GALT) comprises the appendix, Peyer's patches in the ileum and isolated lymphoid follicles in the small and large intestine. GALT is the inductive site for the generation of protective immune responses against enteric pathogens as well as the tolerogenic response to commensal species (19). Interestingly, the appendicectomy of the (inflamed) appendix at a young age has been correlated with a reduced risk of developing UC later in life (20). The underlying mechanism of this protective effect is unknown; however, it strongly suggests that an aberrant immune response in the appendix can predispose to UC. In addition, several reports have described appendiceal inflammation in patients with UC affecting only the distal parts of the colon (21, 22). Based on these studies that suggest that plasma cells, B cells and GALT could be implicated in the development of IBD, we sought to study the B cell populations in blood and GALT of CD and UC patients. In addition, we aimed to study the gut-homing capacity of the different populations in blood by investigating the expression of the β7 subunit of α4β7 integrin in active disease vs. remission. We further looked at how the three main biologic treatments for IBD, the anti-TNF treatment infliximab, the anti-α4β7 integrin treatment vedolizumab, and the anti-IL12/IL23 p40 antibody ustekinumab affect peripheral B cell subsets in IBD. While several B cell aberrations in blood and GALT in IBD were observed, we focus this report on the poorly understood CD27^−^IgD^−^ population that are likely to be unconventional memory B cells (23–27), that we found to be consistently depleted in CD and UC blood, but enriched in GALT.

## Materials and Methods

### Sample Collection

All recruited IBD patients had a confirmed diagnosis of either CD or UC and took part with informed written consent. The study was approved by the local ethics committee (REC 10/H0704 and REC 15/LO/2127). Blood samples were obtained from patients who attended the biologics infusion clinic or the outpatient IBD clinic for a routine review. Controls for the study of blood cells were healthy donors who were age and gender matched to the patients in each group. Endoscopic samples were taken from IBD patients undergoing routine colonoscopies to assess disease activity, and from patients undergoing polypectomy who had no signs of intestinal inflammation, serving as controls. GALT samples were obtained by targeted biopsies of ileal Peyer's patches and the appendiceal orifice. Mononuclear cells from biopsies were isolated using a collagenase digest as previously described (28). Blood samples were collected in sodium heparin tubes and peripheral blood mononuclear cells (PBMCs) were isolated using Ficoll-Paque (GE Healthcare, Amersham, UK) density gradient centrifugation as previously described (28). PBMCs were cryopreserved using fetal bovine serum (FBS)/10% dimethyl sulfoxide (DMSO; both Sigma Aldrich, Gillingham, UK) and stored in the vapor phase of liquid nitrogen until use. An overview of recruited patients is provided in Supplemental Tables 1, 2. For cell sorting experiments blood cones from healthy donors were purchased from NHS Blood and Transplant (Tooting, UK).

### Patient Stratification

We included 10 patients receiving infliximab (8 CD and 2 UC; 55% female, median age 41); six patients receiving vedolizumab (1 CD and 5 UC, 50% female, median age 32), and 14 CD patients receiving ustekinumab (50% female, median age 32). Patients received:

- Intravenous (IV) ustekinumab (Stelara®) 6 mg/kg (induction) and 90 mg subcutaneously every 8 weeks from week 8.
- IV infliximab (Remsima®) 5 mg/kg at week 0, 2, and 6 thereafter every 8 weeks.
- IV vedolizumab (Entyvio®) 300 mg at week 0, 2, and 6 and thereafter every 8 weeks.

Blood samples were prospectively collected pre-treatment (week 0), at 6 weeks (infliximab and vedolizumab) or 8 weeks (ustekinumab). Clinical response/remission was assessed at week 14 (infliximab and vedolizumab) or week 16 (ustekinumab). Clinical remission was defined as Harvey-Bradshaw index (HBI) <5 or partial Mayo score 0–1 at week 14/16 (29, 30). Clinical response was defined as HBI reduction ≥3 or partial Mayo score reduction >/=2 for patients with inactive disease at baseline, or >30% reduction if disease activity index was abnormal (HBI ≥5, partial Mayo ≥2) at week 0. Biological response was defined as a 50% reduction in CRP, if baseline CRP >5 mg/l. Biological remission was defined as CRP <5 mg/l (31).

### Multi-Parameter Flow Cytometry

**Figure 1:**
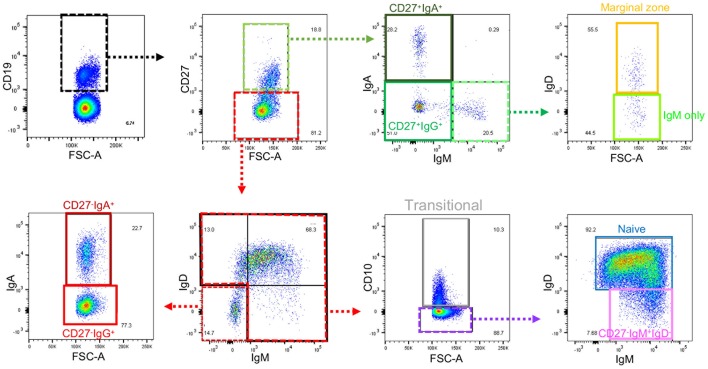
Gating strategy for B cell subsets from blood. Gating strategy to define nine subsets of B cells using the same color code as the gating plan in Supplemental Table 4. Further details of gating prior to the selection of CD19^+^ cells are in Supplemental Figure 1. Gates that define intermediate populations are dotted. Gates that define final populations studied are solid.

Frozen PBMCs from healthy volunteers and IBD patients were thawed, washed and rested for 45 min in RPMI-1640 containing 10% FBS, 100 U/ml penicillin/ 100 μg/ml streptomycin (all Gibco/Thermo Fisher Scientific, Waltham, MA, USA), with DNase I (0.1 mg/ml; from Roche, Welwyn Garden City, UK). Cells were resuspended in PBS prior to live dead staining with Zombie Aqua (Biolegend, San Diego, CA, USA). Cells were then stained with fluorochrome conjugated monoclonal antibodies to CD19, CD27, CD10, IgA, IgD, IgM, and integrin β7 (Supplemental Table 3). Cells were analyzed using a BD Biosciences LSR Fortessa flow cytometer (Wokingham, UK) before determination of B cell populations using the gating strategy shown in Figure 1. Further detail and explanation are provided in Supplemental Figure 1 and Supplemental Table 4. IgG was not used in the flow cytometry panel, but preliminary experiments demonstrated that cells lacking IgM, IgA (and IgD for the CD27^−^ subset) could be reasonably classified as IgG^+^, so this method was used to reduce antibodies in the panel. One of five test experiments is illustrated in Supplemental Figure 2. Although theoretically IgE expressing B cells could have been included, these are known to be very small in number and are difficult to detect due to the possibility of IgE binding to Fc receptors.

### Cell Sorting

Frozen blood cone derived PBMCs were thawed, washed and rested in RPMI-1640 containing 10% FBS, 100 U/ml penicillin/ 100 μg/ml streptomycin, and DNase I (0.1 mg/ml) for 45 min. Cells were stained with DAPI, anti-CD19, CD27, and IgD prior to sorting using a BD Biosciences FACSAria II Cell Sorter. B cells were sorted initially by CD19 expression, with four populations isolated based on CD27 and IgD expression: CD27^+^IgD^−^, CD27^−^IgD^−^, CD27^+^IgD^+^, and CD27^−^IgD^+^ (Supplemental Figure 3A).

### Analysis of Intracellular Cytokines

Unsorted cell suspensions or B cell populations sorted by FACS were suspended in RPMI-1640 containing 10% FBS, 100 U/ml penicillin/ 100 μg/ml streptomycin and seeded into 96 well plates prior to stimulation with 250 ng/ml ionomycin, 50 ng/ml PMA (both Sigma) and GolgiStop (1:1,000; BD Biosciences) for 4 h. Cells were washed and resuspended with PBS prior to live dead staining with Zombie Aqua. Subsequently, cells were washed and fixed with 2% paraformaldehyde, prior to treatment with permeabilization buffer (both eBioscience/Thermo Fisher Scientific). Cells were stained with anti-TNFα (Mab11, BioLegend, 1:50) and anti-IL10 (JES3-19F1, BioLegend, 1:20) and data acquired using a FACS Canto II instrument (BD Biosciences). See Supplemental Figure 4 for gating strategies for analysis of cytokine production by whole CD19^+^ populations from GALT and blood and Supplemental Figure 3 for sorting CD27^+^IgD^−^, CD27^−^IgD^−^, CD27^+^IgD^+^, and CD27^−^IgD^+^ populations and for analysis of cytokine production by cultured sorted cells.

### Mass Cytometry

Cells isolated from healthy control mucosal biopsy samples (*n* = 6) and IBD (UC: *n* = 6 and CD: *n* = 1) were washed and rested in RPMI-1640 10% FBS, 100 U/ml penicillin/100 μg/ml streptomycin and DNase I (0.1 mg/ml) for 30 min. Cell counts were obtained using trypan blue (Sigma) on a Countess II FL cell counter (Thermo Fisher Scientific). From each sample two million live cells were stained with Cell-ID Intercalator-103 Rh (1:500; Fluidigm, San Francisco, CA, USA) for DNA for 15 min. After washing cells were stained with metal-conjugated antibodies (Supplemental Table 5) for 30 min, then washed and fixed in 2% paraformaldehyde overnight at 4°C. Cells were pelleted and frozen in FBS/10% DMSO at −80°C for no longer than 14 days. On the day of the mass cytometry run, cells were thawed and washed prior to incubation with 0.3% saponin with Cell-ID Intercalator-Ir for 20 min to permeabilize and live/dead stain, respectively (32). Cells were then washed with PBS followed by two water washes before being run into a Fluidigm Helios mass cytometry instrument.

### Data Analysis

Flow cytometry data was analyzed in FlowJo software (FlowJo LCC, Ashland, Oregon, USA) to identify nine B cell populations, as described in Figure 1, Supplemental Figures 1, 2, and Supplemental Table 4. The designation of cells that do not express IgM or IgA (or IgD for CD27^−^ cells) as IgG^+^ was based on preliminary experiments Supplemental Figure 2.

Mass cytometry data was analyzed using cloud-based cytometry platform, Cytobank (Santa Clara, CA USA https://mrc.cytobank.org). Bead-based normalization of the individual mass cytometry data was performed with the Normalizer v0.3 software from Garry P. Nolan Laboratory (downloaded from https://github.com/nolanlab/bead-normalization/releases/tag/v0.3). Supplemental Figure 5A shows pre and post-normalization plots. Normalized files were gated as shown in Supplemental Figures 5B,C after uploading onto Cytobank to remove doublets by gating on DNA and removing cells with implausible marker combinations. Data was generated by further subset gating in Cytobank.

### Statistical Analysis

Statistical testing was performed using GraphPad software (La Jolla, CA, USA). Individual groups were compared using Mann-Whitney U non-parametric test. Multiple groups were compared using Kruskal-Wallis ANOVA. A *p* < 0.05 was considered significant. Identifiers of statistical values derived from Kruskal-Wallis ANOVA are identified with red lines and asterisks in the figures. Identifiers of statistical values derived using the Mann-Whitney *U* test are identified with black lines and asterisks in the figures.

## Results

### Altered B Cell Subset Frequencies in Peripheral Blood of IBD Patients

**Figure 2:**
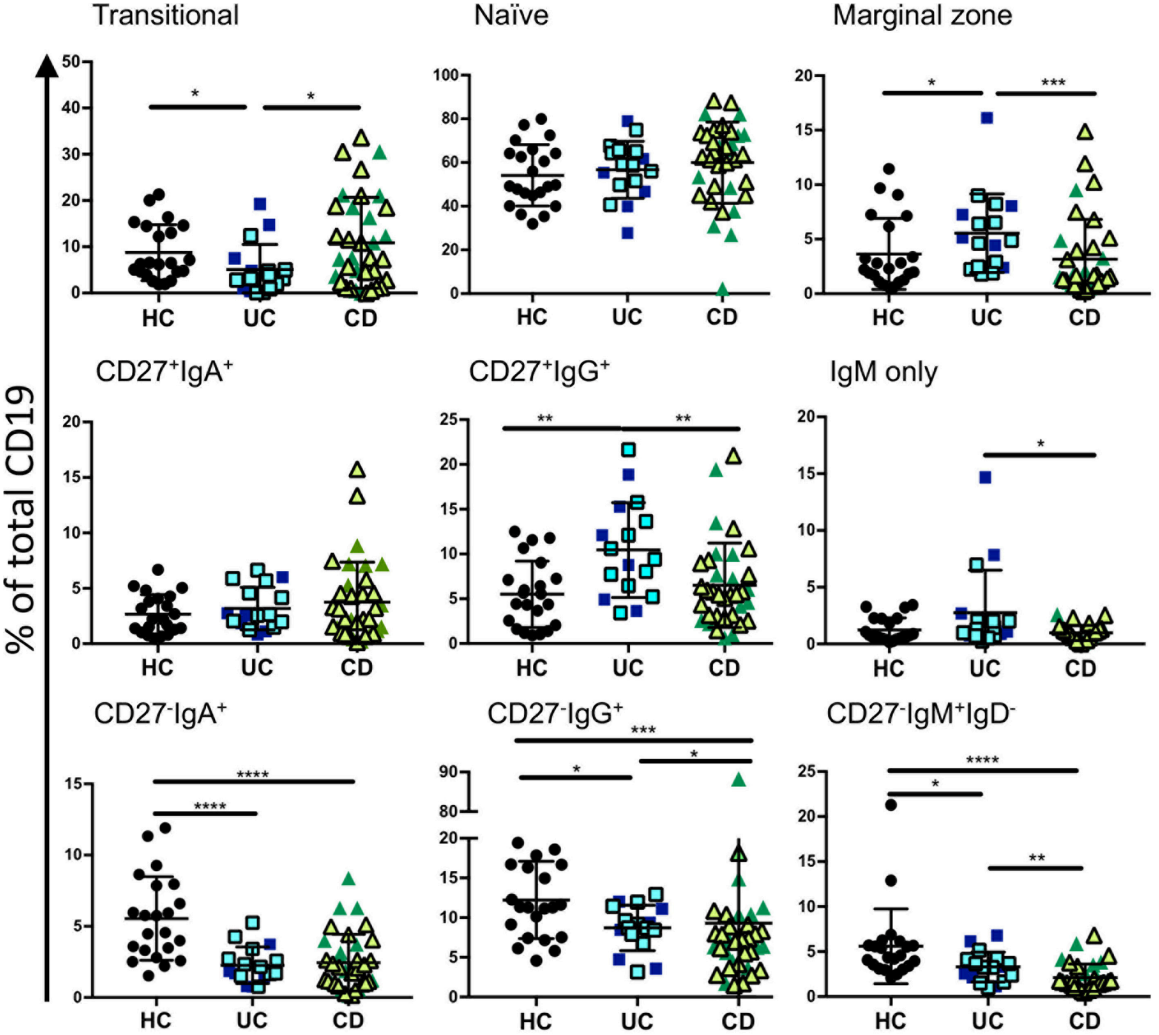
Analysis of B cell subsets in healthy controls and IBD patients. Peripheral blood mononuclear cells from IBD and HC patients were stained using fluorescent antibodies and gated according to the strategy in Figure 1 and Supplemental Table 4. Data is presented as percentage of total CD19 positive cells from each individual HC (*n* = 22), UC patient (*n* = 17), and CD patient (*n* = 35). Patients with UC in are represented with blue symbols. Symbols that are lighter blue with black borders are patients in remission. Patients with CD in are represented with green symbols. Symbols that are lighter green with black borders are patients in remission. Differences between groups were analyzed by Mann-Whitney *U* test where *p* < 0.05 = ^*^, *p* < 0.01 = ^**^, *p* < 0.001 = ^***^, and *p* < 0.0001 = ^****^.

In order to assess relative frequencies of the main B cell subsets in peripheral blood of CD and UC patients, we subjected the PBMCs of patients with various disease activities and treatments (see Supplemental Table 1) to six color flow cytometry. B cell subsets were gated as described in Figure 1, Supplemental Figure 1 and Supplemental Table 4. In UC blood we observed a moderate reduction of transitional B cells (CD27^−^IgM^+^IgD^+^CD10^+^) and an increase in marginal zone B cells (CD27^+^IgM^+^IgD^+^) compared to controls (Figure 2). The frequencies of naïve, CD27^+^ IgA^+^, and CD27^+^IgM^+^ subsets did not differ between IBD and controls (Figure 2). In UC blood, the frequency of CD27^+^IgG^+^ was increased compared to both HC and CD (Figure 2). Interestingly, the most striking difference compared to controls was the overall reduction of CD27^−^IgD^−^ populations expressing IgM, IgA or IgG in the blood of both UC and CD patients (Figure 2).

### Increased Frequencies of CD27^−^IgD^−^ B Cells in IBD GALT

**Figure 3:**
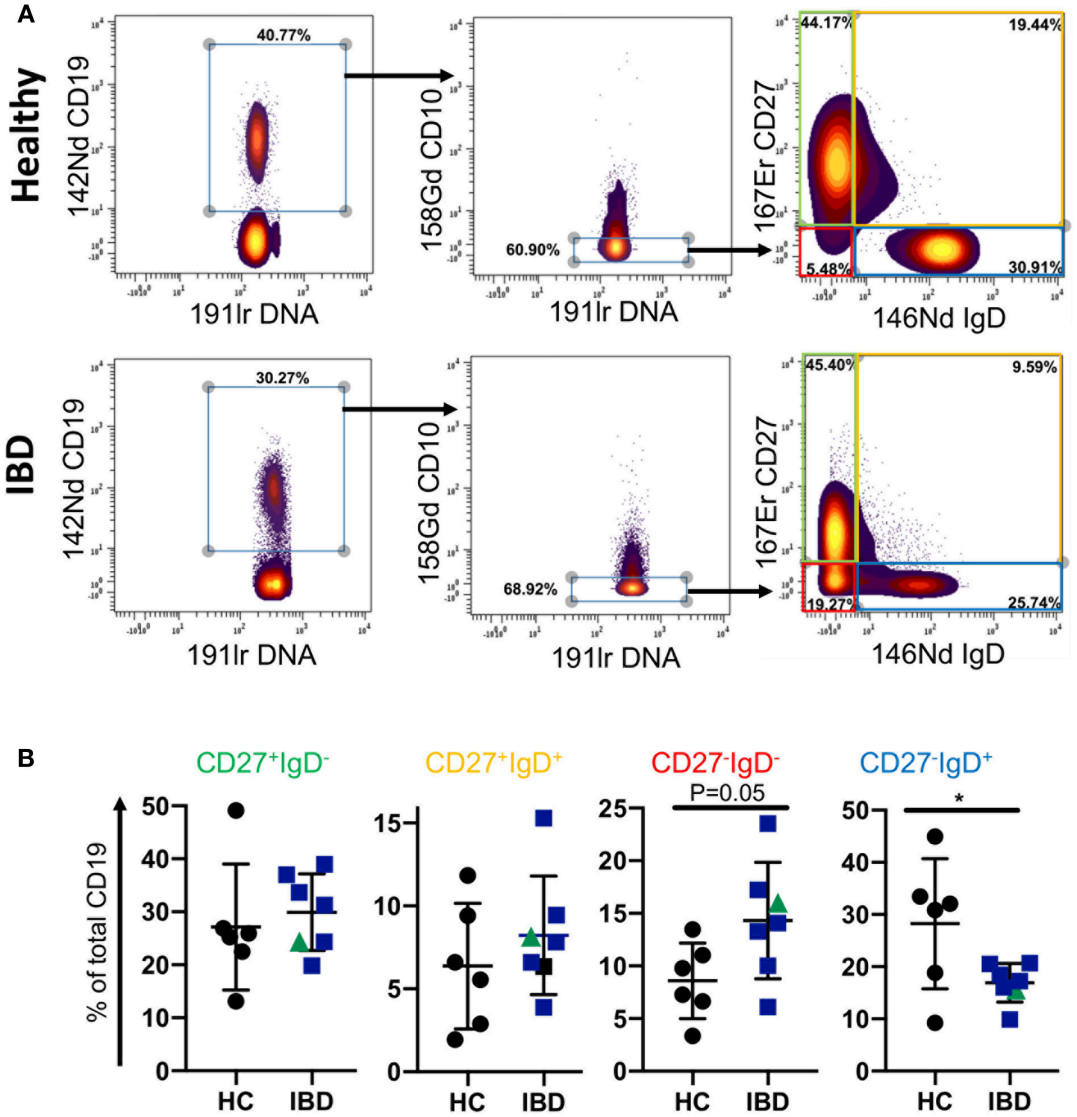
B cell subsets in GALT of healthy control and IBD patients. Data was gated on the Cytobank platform. Following selection of CD19^+^ cells and elimination of CD10^+^ cells (since these would include germinal center cells in tissues and therefore not be equivalent to CD10^+^ subsets in blood), cells were gated according to their expression of CD27 and IgD. Examples of a HC and an IBD sample are illustrated in **(A)**. In **(B)** dotplots represent individually gated data points for HC (*n* = 6), patients with UC (*n* = 6 in blue) and a patient with CD in green, groups together as IBD. Groups were compared by Mann-Whitney *U* test where *p* < 0.05 = ^*^.

Since the proportion of CD27^−^IgD^−^ B cells of total CD19 B cells was reduced in blood of patients with IBD, we asked whether this is also a feature of B cells in GALT of patients with IBD. To answer this specific question, we interrogated a large dataset available in our lab that had been generated by mass cytometric analysis of cells isolated from GALT of healthy individuals and patients with IBD. Following initial normalization and quality control we analyzed the data by manually gating the .fcs files in Cytobank (Supplemental Figure 5, Supplemental Table 4 and Figure 3). We initially gated on CD19^+^ cells and then excluded CD10^+^ cells since these would include germinal center cells that are not comparable with blood. We then analyzed the frequencies of CD27^+^IgD^−^, CD27^−^IgD^−^, CD27^+^IgD^+^, and CD27^−^IgD^+^ B cells and observed an increase in the proportion of CD27^−^IgD^−^ cells and a reduction in CD27^−^IgD^+^ cells in GALT in IBD. This demonstrated that the CD27^−^IgD^−^ B cell subset is not globally depleted in patients with IBD.

### Expression of Gut-Homing Marker Beta7 Integrin on CD27^−^IgD^−^ B Cells

The enrichment of CD27^−^IgD^−^ cells in GALT in IBD, and the reduction of all subsets of this population expressing IgM, IgA, and IgG in blood in IBD, implies that CD27^−^IgD^−^ cells may be recruited from the blood to the gut in IBD. Therefore, we went on to investigate the gut-homing capacity of those cells in blood of UC and CD patients by analyzing the expression of α4β7 integrin (by staining the β7 sub-unit). In addition, we were interested in determining whether this might differ in active disease compared to remission state. For the analysis samples were retrospectively stratified into raised C-reactive peptide (CRP >5 mg/l, indicating active inflammation) and normal CRP (CRP <5 mg/l). Samples were also stratified by a global clinical assessment of the patients being having active disease compared to being in remission.

**Figure 4:**
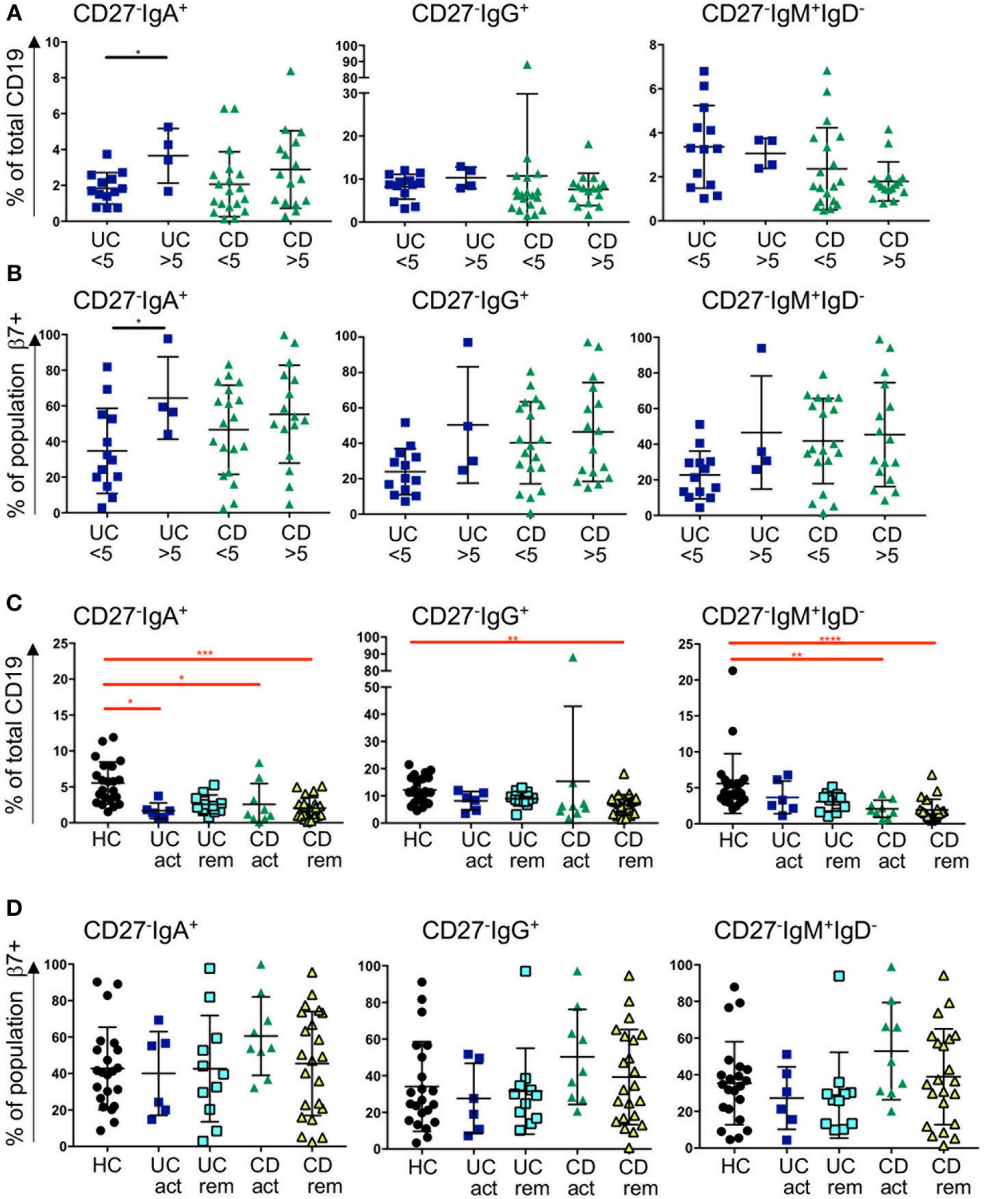
Frequency of blood CD27^−^IgD^−^ subsets and β7 integrin expression in CD and UC patients stratified according to CRP levels and remission status. Three subsets of CD27^−^IgD^−^ B cells (CD27^−^IgA^+^, CD27^−^IgG^+^, and CD27^−^IgM^+^) are presented as percentage of CD19 positive cells in **(A)** and in **(B)** the percentage of individual populations expressing of β7 integrin. For **(A,B)**, UC < 5 *n* = 13, UC > 5 *n* = 4, CD < 5 *n* = 19, CD > 5 *n* = 16; for **(C,D)** HC *n* = 22, UC active *n* = 6, UC remission *n* = 11, CD active *n* = 9 and CD remission *n* = 22. Data is analyzed by Mann-Whitney *U* test (black bars and asterisks) or Kruskal-Wallis ANOVA test (red bars and asterisks) where *p* < 0.05 = ^*^, *p* < 0.01 = ^**^, *p* < 0.001 = ^***^ and *p* < 0.0001 = ^****^.

The frequency of CD27^−^IgA^+^ cells was slightly raised in UC patients with CRP >5 compared to CRP <5 and the expression of β7 integrin increased when CRP was elevated. A similar trend was observed for the CD samples but did not reach significance (Figures 4A,B). In UC with CRP>5, the frequencies of CD27^−^IgG^+^ cells and CD27^−^IgM^+^IgD^−^ cells showed a non-significant trend toward being raised with increased β7 expression. No differences were seen for those two subsets and β7 in CD with a CRP>5 in terms of % positive cells (Figure 4B), however MFI of β7 integrin expression was lower in CD27^−^IgM^+^IgD^−^ cells in patients with CD when CRP was >5 compared to <5 (Supplemental Figure 6). When patients were stratified clinically into either active disease or in remission, we still observed a bias toward reduced proportion of CD27^−^IgD^−^ cells. The reduction of CD27^−^ IgD^−^ was significantly reduced in blood from CD patients compared to HC independently of disease activity (Figure 4C). For the UC samples we observed a non-significant trend of such a reduction. The expression of β7 integrin on CD27- cells in CD blood as either % positive cells or MFI tended to be raised but values did not reach statistical significance (Figure 4D and Supplemental Figure 6).

### Treatment With Biologics has Little Impact on Blood CD27^−^IgD^−^ Cells in IBD

**Figure 5:**
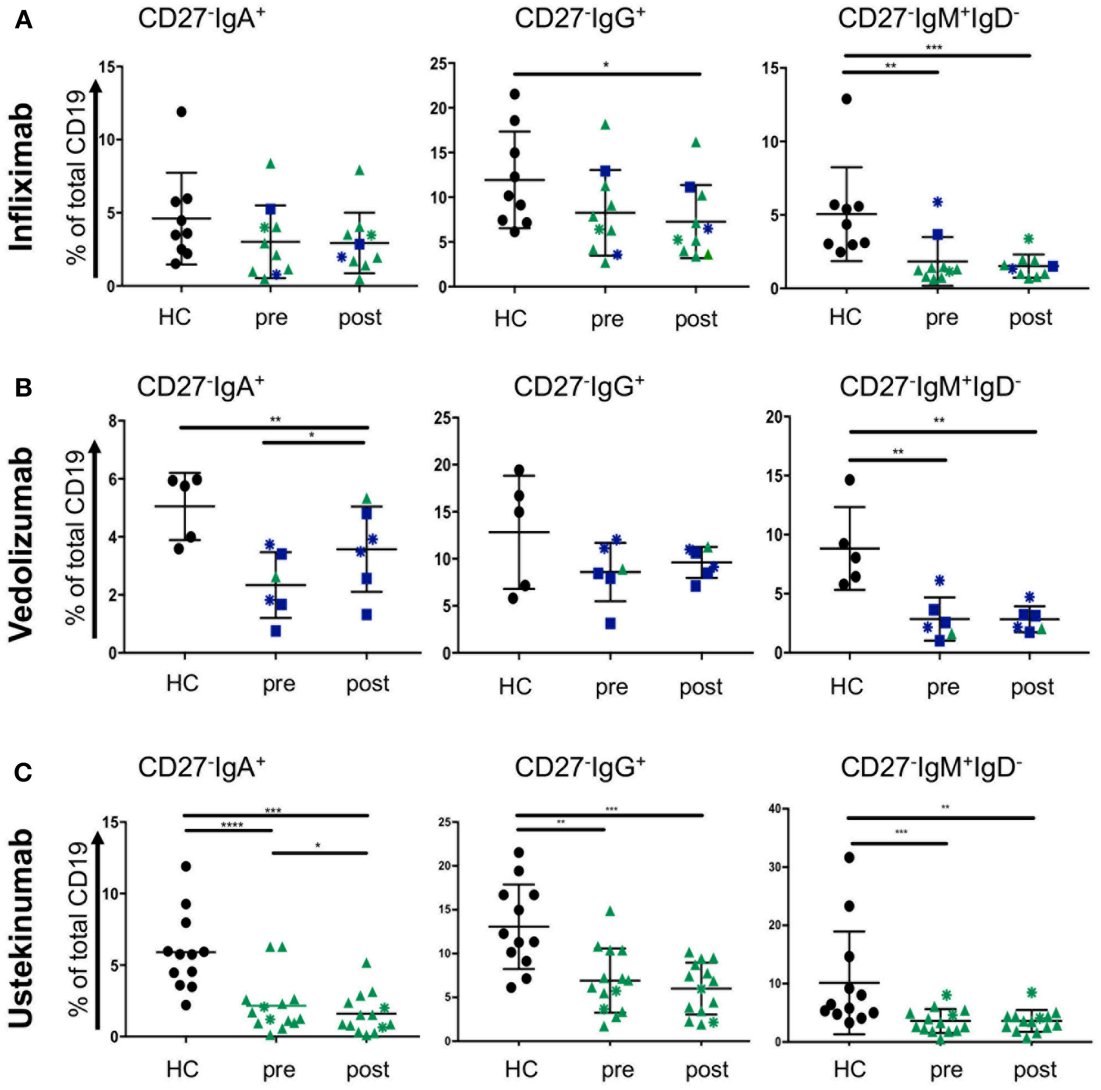
CD27^−^IgD^−^ B cells subsets in blood of CD and UC patients treated with biologics. Patients were treated with either **(A)** infliximab (*n* = 10, HC *n* = 9), **(B)** vedolizumab (*n* = 6, HC *n* = 5), or **(C)** ustekinumab (*n* = 14, HC *n* = 12) with a baseline (pre) and a post-treatment (6 weeks for infliximab and vedolizumab, and 8 weeks for ustekinumab) sample obtained. Subsets of CD27^−^IgD^−^ cells were compared to age and gender matched healthy controls (Dark blue squares: UC patients responsive to treatment; Dark blue asterisks: UC patients non-responsive to treatment; Green triangles: CD patients responsive to treatment; Green asterisks: CD patients non-responsive to treatment). Data was analyzed by Mann-Whitney *U* tests where *p* < 0.05 = ^*^, *p* < 0.01 = ^**^, *p* < 0.001 = ^***^ and *p* < 0.0001 = ^****^.

Treatment escalation in advanced CD and UC cases involves the use of biologic treatments. The most frequently used are the anti-TNF antibody infliximab and the anti-α4β7 antibody vedolizumab for UC and CD. The latter disease can also be treated with the anti-p40 antibody ustekinumab. Little is known how those antibodies affect blood B cells populations. While we did not anticipate a direct effect of these drugs on B cells, we wondered whether the induction of remission and hence improved gut barrier function could have an impact. CD27^−^IgD^−^ cells have been shown to be responsive to bacterial antigens (33). Therefore, we hypothesized that mucosal healing during the course of treatment might affect the proportion of CD27^−^IgD^−^ B cells in the blood stream of IBD patients. Infliximab treatment (sample obtained at week 6; “post”) did not change the frequencies of CD27^−^IgA^+^, CD27^−^IgG^+^, or CD27^−^ IgM^+^ IgD^−^ populations compared to the corresponding sample taken before treatment commenced (Figure 5A). The effect of vedolizumab was also assessed at week 6 (“post”) and, interestingly, only the CD27^−^IgA^+^ population was increased compared to the sample taken before treatment (“pre”; Figure 5B), suggesting that vedolizumab retains this population in the blood by blocking gut homing via α4β7. The CD27^−^IgG^−^, and CD27^−^IgM^+^IgD^−^ populations were lower in IBD blood compared to controls but did not change during treatment (Figure 5B). Ustekinumab treatment mildly lowered the frequency of CD27^−^IgA^+^ cells (week 16; post, compared to treatment start; pre. Figure 5C) but did not affect the frequencies of CD27^−^IgG^+^, or CD27^−^IgM^+^IgD^−^ cells (Figure 5C).

### B Cells in GALT Produce TNFα and IL-10

**Figure 6:**
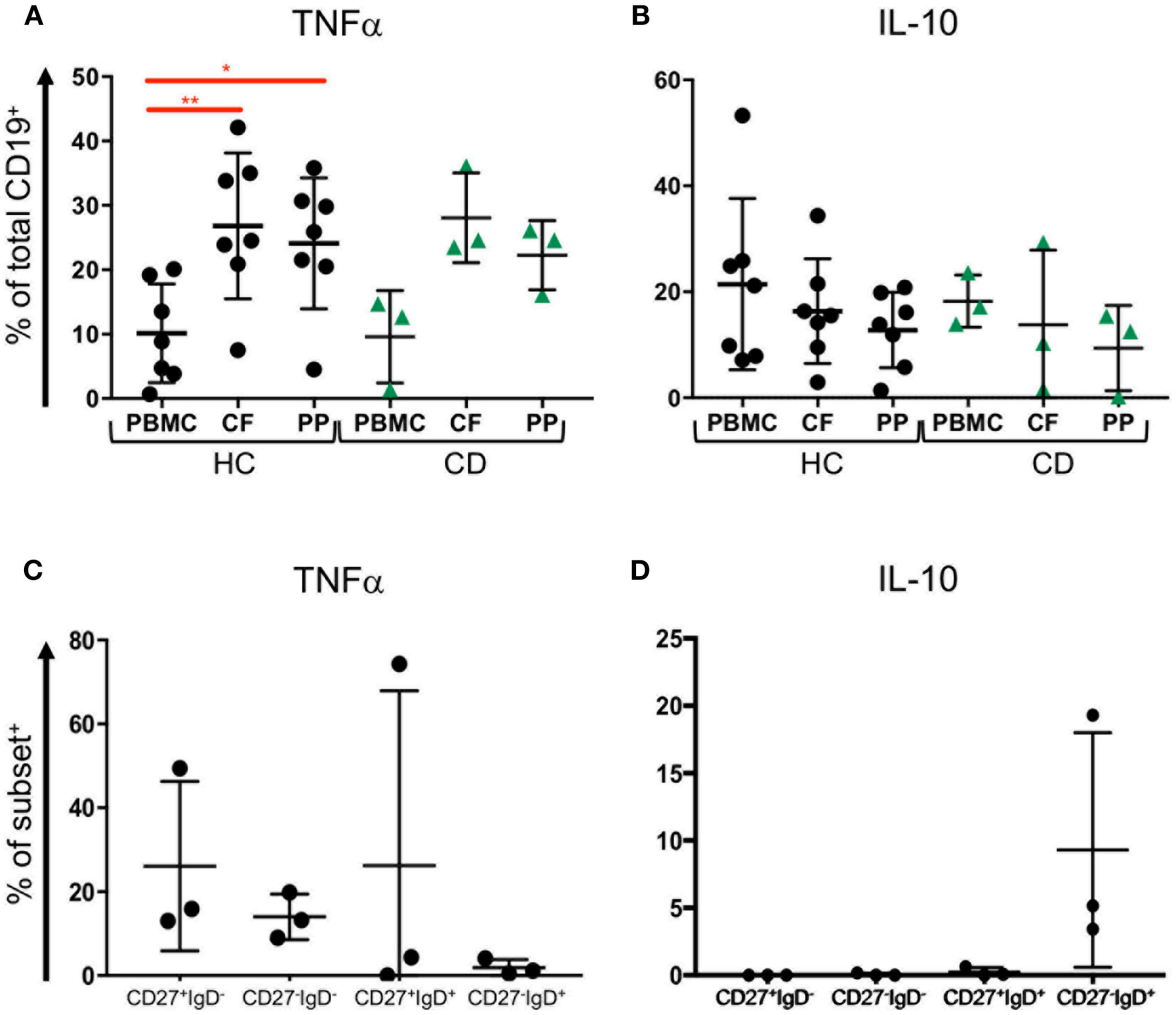
TNFα and IL-10 expression by blood and tissue B cells. Blood (PBMC) and GALT (CF, colonic follicles; PP, Peyer's patches) samples were obtained from HC (*n* = 7) and CD patients (*n* = 3) and isolated CD19^+^ cells were analyzed for **(A)** TNFα and **(B)** IL-10 expression according to the strategy in Supplemental Figure 4. Subsets of B cells were sorted from blood as in Supplemental Figure 3, and expression of **(C)** TNFα and **(D)** IL-10 measured. Data was analyzed by Kruskal Wallis ANOVA test, where *p* < 0.05 = ^*^ and *p* < 0.01 = ^**^.

Since our data show that B cell subsets are represented differently in blood and GALT, we wondered whether those B cells might be capable of producing cytokines such as TNFα and IL-10 (34). We analyzed B cells isolated from blood and two GALT sites: the Peyer's patches in the terminal ileum and colonic follicles in the rectum from the same donors for their production of intracellular TNFα and IL10 (Supplemental Figure 4). Compared to the donor-matched PBMCs, the B cells in healthy GALT showed greater tendency to produce TNFα (~25% compared to 10%). Interestingly, slightly more B cells isolated from colonic follicles produced TNFα than their counterparts isolated from Peyer's patches. B cells in GALT from CD patients also tended to produce more TNFα than the corresponding PBMCs (Figure 6A). In both health and CD, PBMCs and matching GALT B cells were able to produce IL-10 (around 20% of all B cells). There was a non-significant trend for more B cells producing IL-10 in blood compared to GALT (Figure 6B).

To determine if CD27^−^IgD^−^ B cells might differ in cytokine production and therefore might differ in their potential to drive inflammation compared to other subsets, FACS sorted CD27^+^IgD^−^, CD27^−^IgD^−^, CD27^+^IgD^+^, and CD27^−^IgD^+^ B cells underwent intracellular staining and flow cytometry for intracytoplasmic TNFα and IL-10 (Supplemental Figure 3). CD27^+^IgD^−^, CD27^−^IgD^−^, CD27^+^IgD^+^ populations but fewer CD27^−^IgD^+^ B cells had intracytoplasmic TNFα (Figure 6C). In contrast, neither CD27^+^IgD^−^, CD27^−^IgD^−^ nor CD27^+^IgD^+^ cells produced IL10 following stimulation, while between 5 and 20% CD27^−^IgD^+^ cells produced IL-10 (Figure 6D).

## Discussion

In this study we aimed to gain insights into the peripheral B cell subsets in IBD and how those subsets might differ between active disease and the state of remission. We observed a consistent proportionate reduction of CD27^−^IgD^−^ B cells expressing IgM, IgA, and IgG in the blood in IBD and an enrichment of the CD27^−^IgD^−^-subset in GALT in IBD, consistent with recruitment of CD27^−^IgD^−^ B cells from the blood to the gut in IBD. Depletion of CD27^−^IgD^−^ B cells from the blood is seen in patients with both CD and UC even when patients are in remission and when disease activity is low. Although, differences in transitional B cells, marginal zone B cells and CD27^+^IgG^+^ B cells were observed, mainly between UC and controls, this could be in compensation for the relative fall in the CD27^−^IgD^−^ subset, as populations were quantified with CD19^+^ cells remaining at 100% and total B cell counts were not determined. We propose that the major feature of the data is the depletion of CD27^−^IgD^−^ subsets of B cells because this was observed consistently across all isotypes and despite the different gating strategies required for their identification from the blood of patients with UC and CD.

The white blood count in IBD can be altered during a disease flare or lowered by treatments like azathioprine and methotrexate (35). However, we also observed our findings in patients with only mild disease or remission, and most patients were not on treatments that cause lymphopenia, suggesting the observed reduction of CD27^−^IgD^−^ subsets is not directly linked to treatment or a flare.

As we were interested in whether peripheral B cell subsets are affected by disease activity in CD and UC, we stratified patients based on their CRP values (CRP >5 mg/l as a marker of ongoing inflammation) and based on clinical scoring by a clinical gastroenterologist. Interestingly, we observed higher frequencies of CD27^−^IgA^+^ cells and an increase in β7 integrin on those cells in blood of UC patients with raised CRP levels. This was not significant for UC patients stratified clinically (Figure 4). Treatment with the antibody vedolizumab significantly raised the frequency of this subset during treatment (measured at 6 weeks; Figure 5B), suggesting that blood CD27^−^IgA^+^ B cells are retained in blood by vedolizumab which hinders gut homing by blocking α4β7. Interestingly, treatment with infliximab and ustekinumab did not have much effect on blood frequencies of CD27^−^IgD^−^ B cells. We only noticed a mild reduction in CD27^−^ IgA^+^ cells at 8 weeks of ustekinumab treatment (Figure 5C). Whether this finding is of therapeutic relevance remains to be determined.

CD27^−^IgD^−^ B cells comprise ~5% of blood B cells in healthy individuals and are considered to be part of the memory B cell pool because they have mutations in their Ig heavy chain genes consistent with having transited though a germinal center (23–27). However, the role of these cells is not understood. They have been associated with aging and inflammatory diseases including rheumatoid arthritis (RA), systemic lupus erythematosus (SLE) and Alzheimer's disease, in which increased frequencies are present in the blood (36–39). Whilst the cause of the increase is not known, this double negative subset has been described as responsive to anti-inflammatory treatments; anti-TNF therapy in RA reduced the cell frequency (40), and conventional treatment in SLE increased it (36). It is therefore very interesting that the change in frequency of CD27^−^IgD^−^ B cells in IBD is in the opposite direction to that observed in aging and other disease settings, and that we do not observe changes in response to biologic therapies. As is often the case with studies of inflammatory diseases, we cannot rule out the possibility that medication given at some time may have induced the changes we see. However, other inflammatory diseases may share the same therapeutic strategies, suggesting that the reduced frequency of CD27^−^IgD^−^ B cells in IBD is a feature of the condition rather than its treatment.

It has been suggested previously that CD27^−^ memory B cells are associated with intestinal B cell responses (25, 33). They are increased in frequency in healthy GALT compared to other lymphoid tissues (41), CD27^−^IgA^+^ memory cells have been shown to have a distinctive repertoire and a bias toward lambda light chain usage. They are polyspecific and able to bind multiple bacterial species in health (33). CD27^−^IgA^+^ memory B cells have also been reported to be generated by T cell independent immune responses since they are present in the blood of patients with deficient CD40/CD40L interactions (25). The increased frequency of CD27-IgD- B cells we observe in GALT in IBD in our study may be due to local proliferation of this subset in response to local challenge rather than selective recruitment from the blood. On the other hand, and in contrast, it has also been suggested that CD27^−^ memory B cells are part of the normal spectrum of conventional memory B cells since clones of B cells can span the CD27^+^ and CD27^−^ subsets (26). In this case, loss or gain of CD27 from members of a clone may reflect time or tissue context rather than lineage or clone specificity.

Our data linking CD27^−^IgD^−^ B cells with intestinal inflammation and their relative depletion from the blood and enhanced frequencies in gut would be consistent with the concept that they are a distinct population with a role in antibacterial immunity. The mucosal barrier is known to be disrupted to varying degrees in IBD so that the local bacterial challenge is likely to be higher (42). UC and CD have markedly different pathogeneses and are impacted by different genetic predispositions and environmental drivers. It is interesting therefore that they share the feature of depletion of CD27^−^ B cells from the blood. This suggests that recruitment of CD27^−^ B cells to the gut is more likely a response to an intestinal challenge rather than a feature of the disease process *per se*. This is potentially also the case in quiescent IBD as persistent changes to epithelial barrier function have been described in UC patients who had no mucosal defects (43, 44).

Since our data shows that GALT B cells and blood B cells, including CD27^−^IgD^−^ memory cells, are able to produce TNFα, we suggest that increased recruitment of these cells to the gut might contribute to the inflammatory milieu in IBD.

## Author Contributions

JS and AV designed the study and wrote the manuscript. CP performed flow cytometric analysis and mass cytometry. NZ, TT, and JaS performed experimental design, mass cytometry, data analysis, and data visualization. MC performed sample preparation and flow cytometry. WG and KC analyzed B cell cytokine production. KK, AV, JeS, and LL provided clinical samples and stratified patients by treatment response. All authors contributed to the final manuscript.

### Conflict of Interest Statement

The authors declare that the research was conducted in the absence of any commercial or financial relationships that could be construed as a potential conflict of interest. The handling Editor declared a past co-authorship with one of the authors AV.

## Abbreviations

IBD: inflammatory bowel disease
UC: ulcerative colitis
CD: Crohn's disease
HC: healthy control
CRP: C-reactive peptide
GALT: gut-associated lymphoid tissue.

## References

[1] Xavier RJ Podolsky DK. Unravelling the pathogenesis of inflammatory bowel disease. Nature. (2007) 448:427–34. 10.1038/nature06005 17653185

[2] Murdoch TB Xu W Stempak JM Landers C Targan SR Rotter JI. Pattern recognition receptor and autophagy gene variants are associated with development of antimicrobial antibodies in Crohn's disease. Inflamm Bowel Dis. (2012) 18:1743–8. 10.1002/ibd.22884 22275320 PMC3418471

[3] de Souza HS Fiocchi C. Immunopathogenesis of IBD: current state of the art. Nat Rev Gastroenterol Hepatol. (2016) 13:13–27. 10.1038/nrgastro.2015.186 26627550

[4] Andoh A Imaeda H Aomatsu T Inatomi O Bamba S Sasaki M. Comparison of the fecal microbiota profiles between ulcerative colitis and Crohn's disease using terminal restriction fragment length polymorphism analysis. J Gastroenterol. (2011) 46:479–86. 10.1007/s00535-010-0368-4 21253779

[5] Khan N Trivedi C Shah Y Cole E Lewis J Yang YX The natural history of newly diagnosed ulcerative colitis in patients with concomitant primary sclerosing cholangitis. Inflamm Bowel Dis. (2018) 24, 2062–7. 10.1093/ibd/izy106 29697792

[6] Abraham C Cho JH. Inflammatory bowel disease. N Engl J Med. (2009) 361:2066–78. 10.1056/NEJMra0804647 19923578 PMC3491806

[7] Horio Y Uchino M Bando T Chohno T Sasaki H Hirata A. Rectal-sparing type of ulcerative colitis predicts lack of response to pharmacotherapies. BMC Surg. (2017) 17:59. 10.1186/s12893-017-0255-5 28526076 PMC5437574

[8] Danese S Angelucci E Malesci A Caprilli R. Biological agents for ulcerative colitis: hypes and hopes. Med Res Rev. (2008) 28:201–18. 10.1002/med.20103 17464967

[9] Scribano ML. Vedolizumab for inflammatory bowel disease: from randomized controlled trials to real-life evidence. World J Gastroenterol. (2018) 24:2457–67. 10.3748/wjg.v24.i23.2457 29930467 PMC6010939

[10] Kobayashi T Okamoto S Hisamatsu T Kamada N Chinen H Saito R. IL23 differentially regulates the Th1/Th17 balance in ulcerative colitis and Crohn's disease. Gut. (2008) 57:1682–9. 10.1136/gut.2007.135053 18653729

[11] Caprioli F Bose F Rossi RL Petti L Vigano C Ciafardini C. Reduction of CD68+ macrophages and decreased IL-17 expression in intestinal mucosa of patients with inflammatory bowel disease strongly correlate with endoscopic response and mucosal healing following infliximab therapy. Inflamm Bowel Dis. (2013) 19:729–39. 10.1097/MIB.0b013e318280292b 23448791

[12] Bain CC Mowat AM. Macrophages in intestinal homeostasis and inflammation. Immunol Rev. (2014) 260:102–17. 10.1111/imr.12192 24942685 PMC4141699

[13] Rovedatti L Kudo T Biancheri P Sarra M Knowles CH Rampton DS. Differential regulation of interleukin 17 and interferon gamma production in inflammatory bowel disease. Gut. (2009) 58:1629–36. 10.1136/gut.2009.182170 19740775

[14] Kamada N Hisamatsu T Honda H Kobayashi T Chinen H Takayama T. TL1A produced by lamina propria macrophages induces Th1 and Th17 immune responses in cooperation with IL-23 in patients with Crohn's disease. Inflamm Bowel Dis. (2010) 16:568–75. 10.1002/ibd.21124 19834969

[15] Bennike TB Carlsen TG Ellingsen T Bonderup OK Glerup H Bogsted M. Neutrophil extracellular traps in ulcerative colitis: a proteome analysis of intestinal biopsies. Inflamm Bowel Dis. (2015) 21:2052–67. 10.1097/MIB.0000000000000460 25993694 PMC4603666

[16] Thoree VC Golby SJ Boursier L Hackett M Dunn-Walters DK Sanderson JD. Related IgA1 and IgG producing cells in blood and diseased mucosa in ulcerative colitis. Gut. (2002) 51:44–50. 10.1136/gut.51.1.44 12077090 PMC1773274

[17] Uo M Hisamatsu T Miyoshi J Kaito D Yoneno K Kitazume MT. Mucosal CXCR4+ IgG plasma cells contribute to the pathogenesis of human ulcerative colitis through FcgammaR-mediated CD14 macrophage activation. Gut. (2013) 62:1734–44. 10.1136/gutjnl-2012-303063 23013725

[18] Virk R Shinagare S Lauwers GY Yajnik V Stone JH Deshpande V. Tissue IgG4-positive plasma cells in inflammatory bowel disease: a study of 88 treatment-naive biopsies of inflammatory bowel disease. Mod Pathol. (2014) 27:454–9. 10.1038/modpathol.2013.121 23929268

[19] Boursier L Gordon JN Thiagamoorthy S Edgeworth JD Spencer J Human intestinal IgA response is generated in the organized gut-associated lymphoid tissue but not in the lamina propria. Gastroenterology. (2005) 128:1879–89. 10.1053/j.gastro.2005.03.047 15940623

[20] Frisch M Pedersen BV Andersson RE. Appendicitis, mesenteric lymphadenitis, and subsequent risk of ulcerative colitis: cohort studies in Sweden and Denmark. BMJ. (2009) 338:b716. 10.1136/bmj.b716 19273506 PMC2659291

[21] Naves JE Lorenzo-Zuniga V Marin L Manosa M Oller B Moreno V. Long-term outcome of patients with distal ulcerative colitis and inflammation of the appendiceal orifice. J Gastrointestin Liver Dis. (2011) 20:355–8. 10.6018/red/45/6 22187699

[22] Park SH Loftus EV Jr Yang SK. Appendiceal skip inflammation and ulcerative colitis. Dig Dis Sci. (2014) 59:2050–7. 10.1007/s10620-014-3129-z 24705639

[23] Fecteau JF Cote G Neron S. A new memory CD27-IgG+ B cell population in peripheral blood expressing VH genes with low frequency of somatic mutation. J Immunol. (2006) 177:3728–36. 10.4049/jimmunol.177.6.3728 16951333

[24] Wei C Anolik J Cappione A Zheng B Pugh-Bernard A Brooks J. A new population of cells lacking expression of CD27 represents a notable component of the B cell memory compartment in systemic lupus erythematosus. J Immunol. (2007) 178:6624–33. 10.4049/jimmunol.178.10.6624 17475894

[25] Berkowska MA Driessen GJ Bikos V Grosserichter-Wagener C Stamatopoulos K Cerutti A. Human memory B cells originate from three distinct germinal center-dependent and -independent maturation pathways. Blood. (2011) 118:2150–8. 10.1182/blood-2011-04-345579 21690558 PMC3342861

[26] Wu Y-CB Kipling D Dunn-Walters DK. The Relationship between CD27 negative and positive B cell populations in human peripheral blood. Front Immunol. (2011) 2:81. 10.3389/fimmu.2011.00081 22566870 PMC3341955

[27] Clavarino G Delouche N Vettier C Laurin D Pernollet M Raskovalova T. Novel strategy for phenotypic characterization of human b lymphocytes from precursors to effector cells by flow cytometry. PLoS ONE. (2016) 11:e0162209. 10.1371/journal.pone.0162209 27657694 PMC5033467

[28] Vossenkamper A Hundsrucker C Page K van Maurik A Sanders TJ Stagg AJ. A CD3-specific antibody reduces cytokine production and alters phosphoprotein profiles in intestinal tissues from patients with inflammatory bowel disease. Gastroenterology. (2014) 147:172–83. 10.1053/j.gastro.2014.03.049 24704524

[29] Harvey RF Bradshaw JM. A simple index of Crohn's-disease activity. Lancet. (1980) 1:514. 6102236 10.1016/s0140-6736(80)92767-1

[30] Schroeder KW Tremaine WJ Ilstrup DM. Coated oral 5-aminosalicylic acid therapy for mildly to moderately active ulcerative colitis. A randomized study. N Engl J Med. (1987) 317:1625–9. 10.1056/NEJM198712243172603 3317057

[31] Vermeire S Van Assche G Rutgeerts P. The role of C-reactive protein as an inflammatory marker in gastrointestinal diseases. Nat Clin Pract Gastroenterol Hepatol. (2005) 2:580. 10.1038/ncpgasthep0359 16327837

[32] Sumatoh HR Teng KW Cheng Y Newell EW. Optimization of mass cytometry sample cryopreservation after staining. Cytometry A. (2017) 91:48–61. 10.1002/cyto.a.23014 27798817

[33] Berkowska MA Schickel JN Grosserichter-Wagener C de Ridder D Ng YS van Dongen JJ. Circulating human CD27-IgA+ memory B cells recognize bacteria with polyreactive Igs. J Immunol. (2015) 195:1417–26. 10.4049/jimmunol.1402708 26150533 PMC4595932

[34] Lighaam LC Unger PA Vredevoogd DW Verhoeven D Vermeulen E Turksma AW. *In vitro*-induced human IL-10(+) B cells do not show a subset-defining marker signature and plastically co-express IL-10 with pro-inflammatory Cytokines. Front Immunol. (2018) 9:1913. 10.3389/fimmu.2018.01913 30258433 PMC6143818

[35] Meijer B Wilhelm AJ Mulder CJJ Bouma G van Bodegraven AA de Boer NKH. Pharmacology of thiopurine therapy in inflammatory bowel disease and complete blood cell count outcomes: a 5-year database study. Ther Drug Monit. (2017) 39:399–405. 10.1097/FTD.0000000000000414 28489727 PMC5538301

[36] Rodriguez-Bayona B Ramos-Amaya A Perez-Venegas JJ Rodriguez C Brieva JA. Decreased frequency and activated phenotype of blood CD27 IgD IgM B lymphocytes is a permanent abnormality in systemic lupus erythematosus patients. Arthritis Res Ther. (2010) 12:R108. 10.1186/ar3042 20525218 PMC2911899

[37] Bulati M Buffa S Martorana A Candore G Lio D Caruso C. Trafficking phenotype and production of granzyme B by double negative B cells (IgG(+)IgD(-)CD27(-)) in the elderly. Exp Gerontol. (2014) 54:123–9. 10.1016/j.exger.2013.12.011 24389059

[38] Bulati M Buffa S Martorana A Gervasi F Camarda C Azzarello DM. Double negative (IgG+IgD-CD27-) B cells are increased in a cohort of moderate-severe Alzheimer's disease patients and show a pro-inflammatory trafficking receptor phenotype. J Alzheimers Dis. (2015) 44:1241–51. 10.3233/JAD-142412 25408215

[39] Tony HP Roll P Mei HE Blumner E Straka A Gnuegge L. Combination of B cell biomarkers as independent predictors of response in patients with rheumatoid arthritis treated with rituximab. Clin Exp Rheumatol. (2015) 33:887–94. 26517829

[40] Moura RA Quaresma C Vieira AR Goncalves MJ Polido-Pereira J Romao VC. B-cell phenotype and IgD-CD27- memory B cells are affected by TNF-inhibitors and tocilizumab treatment in rheumatoid arthritis. PLoS ONE. (2017) 12:e0182927. 10.1371/journal.pone.0182927 28886017 PMC5590747

[41] Zhao Y Uduman M Siu JHY Tull TJ Sanderson JD Wu YB. Spatiotemporal segregation of human marginal zone and memory B cell populations in lymphoid tissue. Nat Commun. (2007) 9:3857. 10.1038/s41467-018-06089-1 30242242 PMC6155012

[42] McDonnell M Liang Y Noronha A Coukos J Kasper DL Farraye FA. Systemic Toll-like receptor ligands modify B-cell responses in human inflammatory bowel disease. Inflamm Bowel Dis. (2011) 17:298–307. 10.1002/ibd.21424 20806343

[43] Vivinus-Nebot M Frin-Mathy G Bzioueche H Dainese R Bernard G Anty R. Functional bowel symptoms in quiescent inflammatory bowel diseases: role of epithelial barrier disruption and low-grade inflammation. Gut. (2014) 63:744–52. 10.1136/gutjnl-2012-304066 23878165

[44] Dotti I Mora-Buch R Ferrer-Picon E Planell N Jung P Masamunt MC. Alterations in the epithelial stem cell compartment could contribute to permanent changes in the mucosa of patients with ulcerative colitis. Gut. (2017) 66:2069–79. 10.1136/gutjnl-2016-312609 27803115 PMC5749340

