# Supplementary material for: Reduced CD27−IgD− B Cells in Blood and Raised CD27−IgD− B Cells in Gut-Associated Lymphoid Tissue in Inflammatory Bowel Disease
Source: Front Immunol. 2019 Mar 5;10:361. doi: 10.3389/fimmu.2019.00361 (PMC6411645; doi:10.3389/fimmu.2019.00361)
Supplement: Supplementary file 1 [file Data_Sheet_1.pdf]

Supplemental Table 1

	HC	UC	CD
Male	9	8	19
Female	13	9	16
Age	43.86±13.79	33.47±13.05	38.77±13.69
CRP (mg/L)	-	3.64±6.11	8.8±12.3
Active disease	-	6	9
Remission	-	11	22
Infliximab (Remsima)	-	2	8
Vedolizumab (Entyvio)	-	5	1
Ustekinumab (Stelara)	-	-	16
Corticosteroid	-	4	6
DMARD	-	5	8

Supplemental Table 1. Characteristics and medication of patients enrolled in the study of blood cells. DMARD: Disease-modifying anti-rheumatic drugs.

Supplemental Table 2

	**HC**(mc)	**HC**(c)	**UC**(mc)	**CD**(mc)	**CD**(c)
Male	4	2	3	-	2
Female	2	5	3	1	1
Age (average and range)	60(47-71)	45(25-80)	46(30-75)	45	34(31-36)
Corticosteroid	-	-	2	1	nd
Immunosuppressant	-	-	4	-	nd
DMARD	-	-	2	-	nd

Supplemental Table 2. Characteristics of patients enrolled in the study of cells isolated from biopsies. DMARD: Disease-modifying anti-rheumatic drugs. nd= no data available. (mc) = biopsies used for mass cytometry. (c) = biopsies used for analysis of cytokine production by B cells in GALT.

Supplemental Table 3

Manufacturer	Antibody	Clone	Concentration
Miltenyibiotec	IgA PE	IS11-8E10	0.5mg/ml
Biolegend	IgD APC-Cy7	IA6-2	200μg/ml
Biolegend	IgM BV605	MHM-88	160μg/ml
Biolegend	β7 FITC	FIB504	0.5mg/ml
Biolegend	CD10 BV421	HI10a	25μg/ml
Biolegend	CD19 PerCP Cy5.5	HIB19	200μg/ml
Biolegend	CD27 BV711	M-T271	50μg/ml
Biolegend	IL10 APC	JES3-19F1	20μg/ml
Biolegend	TNF-α Pacific Blue	MAb11	0.5mg/ml
Biolegend	Mouse IgG1 κ, BV711	MOPC-21	100μg/ml
BD Biosciences	Mouse IgG1 κ, PerCP Cy5.5	MOPC-21	0.2mg/ml
Biolegend	Mouse IgG1 κ, APC	MOPC-21	0.2mg/ml
Biolegend	Rat IgG2a, κ FITC	RTK2759	0.5mg/ml
BD Pharmingen	Mouse IgG1 κ PE	MOPC-21	0.2mg/ml

Supplemental Table 3. Antibodies used for flow cytometry.

Supplemental Table 4

Population description (out of total CD19+)	Population interpretation	Flow criteria for blood	Mass cytometry gating criteria
Transitional	Transitional	$CD27^{-}IgM^{+}IgD^{+}CD10^{+}$	^$CD27^{-}CD10^{+}$
Naive	Naive	$CD27^{-}IgM^{+}IgD^{+}CD10^{-}$	$CD10^{-}CD27^{-}IgD^{+}$
Marginal zone	Marginal zone	$CD27^{+}IgM^{+}IgD^{+}$	$CD10^{-}CD27^{+}IgD^{+}$
$CD27^{+}IgA^{+}$	Classical IgA memory	$CD27^{+}IgA^{+}$	$CD10^{-}CD27^{+}IgD^{-}$
*$CD27^{+}IgG^{+}$	Classical IgG memory	$CD27^{+}IgA^{-}IgM^{-}$	$CD10^{-}CD27^{+}IgD^{-}$
IgM only	Mixture of marginal zone and memory B cells	$CD27^{+}IgM^{+}IgD^{-}$	$CD10^{-}CD27^{+}IgD^{-}$
$CD27^{-}IgA^{+}$	Non-classical IgA memory	$CD27^{-}IgM^{-}IgD^{-}IgA^{+}$	$CD10^{-}CD27^{-}IgD^{-}$
*$CD27^{-}IgG^{+}$	Non-classical IgG memory	$CD27^{-}IgM^{-}IgD^{-}IgA^{-}$	$CD10^{-}CD27^{-}IgD^{-}$
$CD27^{-}IgM^{+}IgD^{-}$	Non-classical IgM memory	$CD27^{-}IgM^{+}CD10^{-}IgD^{-}$	$CD10^{-}CD27^{-}IgD^{-}$
Germinal centre		Not applicable	^$CD27^{+}CD10^{+}$

Supplemental Table 4. B cell subset gating and population descriptions. B cell subsets are colour coded to cross reference with **Figure 1** and **Figure 3**. *Preliminary data justifying the designation of $IgM^{-}IgA^{-}$ (IgD^{-}) cells as IgG^{+} is shown in **Supplemental Figure 2**. ^Note that the $CD19^{+}CD10^{+}$ populations were not used for analysis of subsets according to expression of CD27 and IgD because the majority of $CD10^{+}$ cells in the gut are germinal centre cells that have no equivalent in blood.

Supplemental Table 5

Antibody	Clone	Metal Tag	Dilution	Supplier
Anti-Human CD10	HI10a	158Gd	1:200	Fluidigm
Anti-Human CD11c	Bu15	147Sm	1:200	Fluidigm
Anti-Human CD127 (IL-7Ra)	A019D5	143Nd	1:200	Fluidigm
Anti-Human CD138 (Syndecan-1)	DL-101	150Nd	1:200	Fluidigm
Anti-Human CD185 (CXCR5)	RF8B2	153Eu	1:200	Fluidigm
Anti-Human CD19	HIB19	142Nd	1:200	Fluidigm
Anti-Human CD197 (CCR7)	G043H7	159Tb	1:200	Fluidigm
Anti-Human CD20	2H7	171Yb	1:200	Fluidigm
Anti-Human CD24	ML5	166Er	1:100	Fluidigm
Anti-Human CD25	2A3	169Tm	1:100	Fluidigm
Anti-Human CD268 (BAFFR)	11C1	155Gd	1:200	Fluidigm
Anti-Human CD27	L128	167Er	1:200	Fluidigm
Anti-Human CD274 (PDL1)	29E.2A3	175Lu	1:200	Fluidigm
Anti-Human CD278 (ICOS)	C398.4A	151Eu	1:200	Fluidigm
Anti-Human CD279 (PD-1)	EH12.2H7	174Yb	1:200	Fluidigm
Anti-Human CD28	CD28.2	160Gd	1:200	Fluidigm
Anti-Human CD3	UCHT1	154Sm	1:200	Fluidigm
Anti-Human CD38	HIT2	144Nd	1:200	Fluidigm
Anti-Human CD4	RPA-T4	176Yb	1:200	Fluidigm
Anti-Human CD45	HI30	89Y	1:200	Fluidigm
Anti-Human CD45RB	MEM-55	145Nd	1:100	Fluidigm
Anti-Human CD45RO	UCHL1	149Sm	1:200	Fluidigm
Anti-Human CD8	SK1	168Er	1:200	Fluidigm
Anti-Human CD80/B7-1	2D10.4	161Dy	1:200	Fluidigm
Anti-Human CD86/B7.2	IT2.2	156Gd	1:200	Fluidigm
Anti-Human IgD	IA6-2	146Nd	1:200	Fluidigm
Anti-Human IgM	MHM-88	172Yb	1:100	Fluidigm
Goat Anti-Human IgA (polyclonal)	-	148Nd	1:200	Fluidigm
Goat Anti-human IgG (polyclonal)	-	141Pr	1:100	Fluidigm
Anti-human CD40	5C3	162Dy	1:200	Biolegend
Anti-human CD154 (CD40L)	24-31	152Sm	1:200	Biolegend
Anti-human CD269 (BCMA)	19F2	173Yb	1:100	Biolegend
Anti-human CD267 (TACI)	1A1	164Dy	1:200	Biolegend
Anti-human CD307d (FcRL4)	413D12	170Er	1:200	Biolegend
Anti-human CD180 (RP105)	MHR73-11	163Dy	1:200	Biolegend
Anti-human CD307e (FcRL5)	509F6	165Ho	1:200	Biolegend

Supplemental Table 5. Antibodies used for mass cytometry.

Supplemental Figure 1

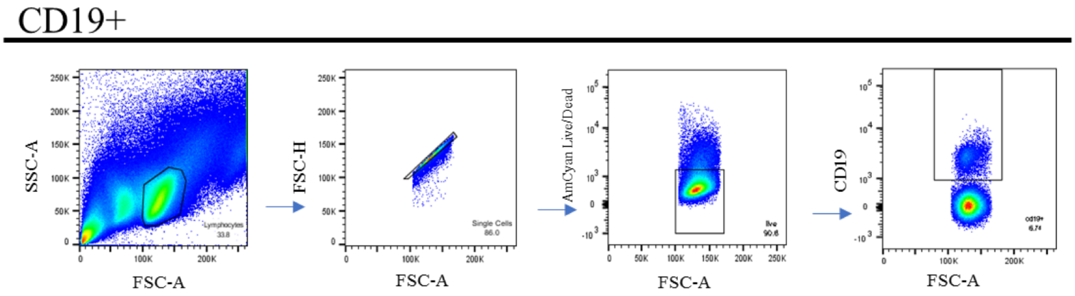

Supplemental Figure 1. Details of gating preceding the gating of B cell subsets from blood illustrated in **Figure 1**.

Supplemental Figure 2

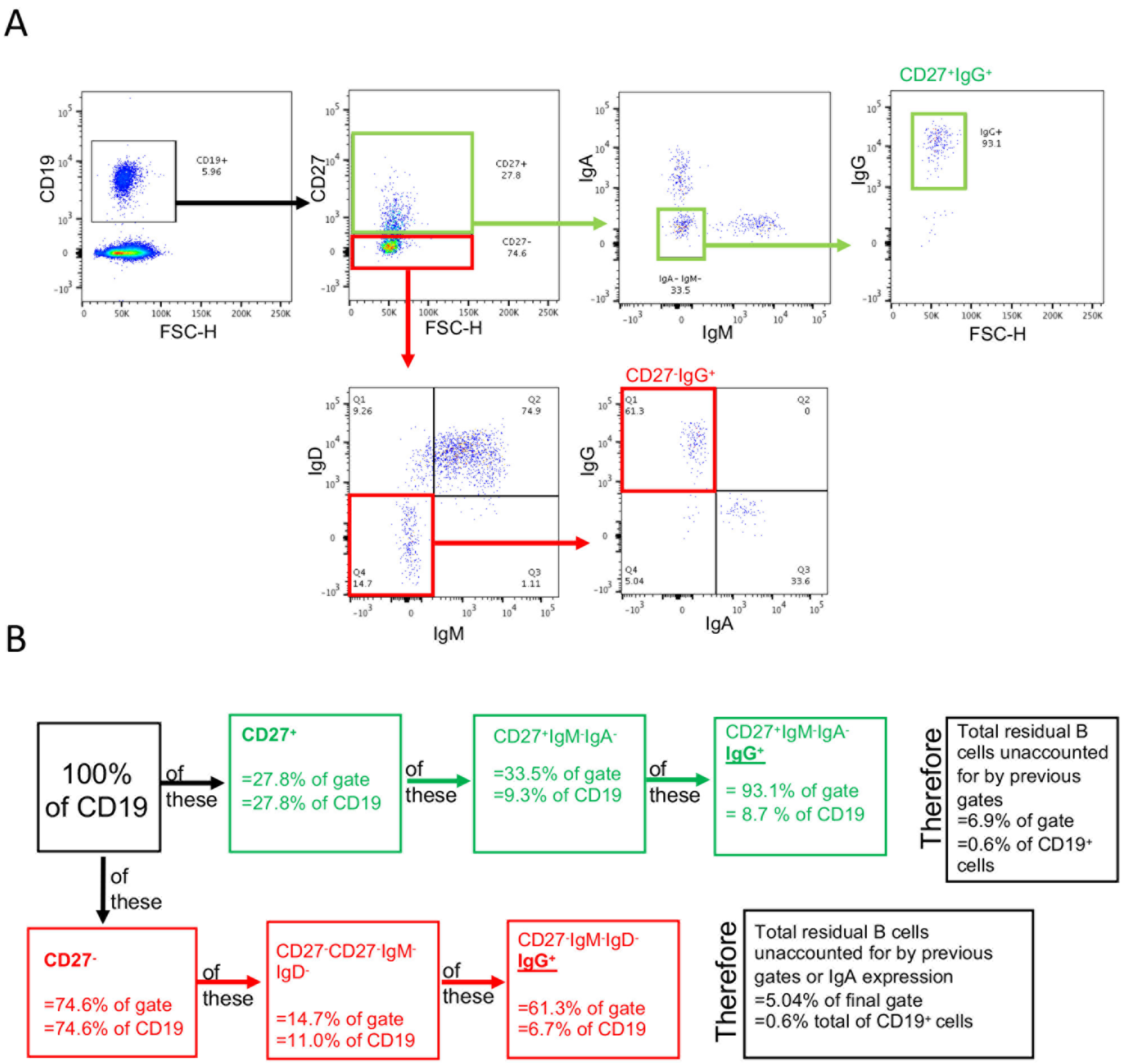

Supplemental Figure 2. Gating of IgG$^+$ cells. Preliminary experiments demonstrated that IgM$^-$IgA$^-$ B cells were almost exclusively IgG$^+$. For this reason IgG was not used in the flow panel and IgM$^-$IgA$^-$(IgD$^-$) cells are referred to as IgG$^+$ in the manuscript for simplicity. A. An example of gating strategy that includes IgG. B. a flow chart linked to the numbers in the quadrants in A. Showing that by including all isotypes in the gating panel only approximately 1% of CD19$^+$ cells cannot be accounted for and these are split evenly between those that originated from the CD27$^+$ gate and the CD27$^-$ gate.

Supplemental Figure 3

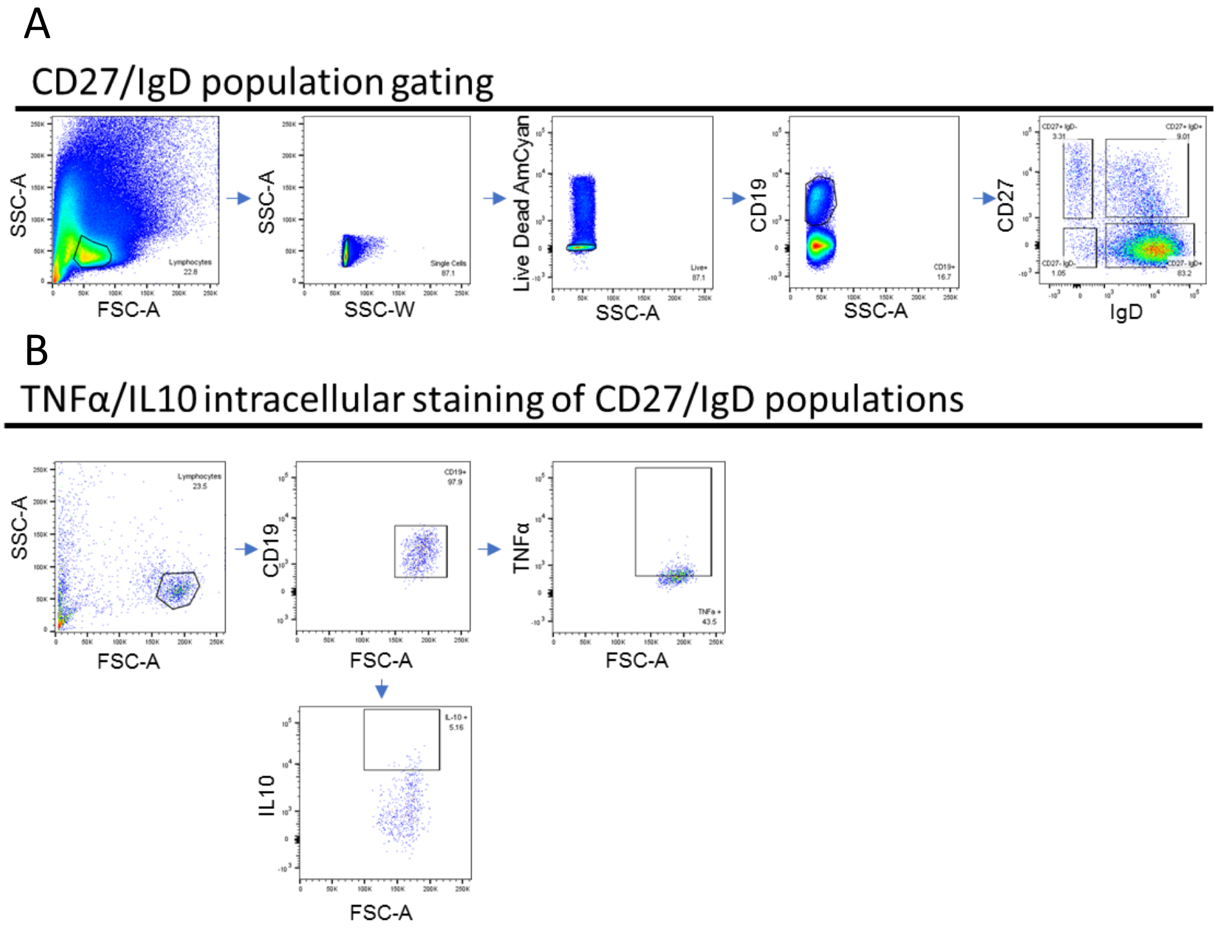

Supplemental Figure 3. Analysis of cytokine production by sorted B cell subsets. A. Scheme for gating of CD27^{+}IgD^{-}, CD27^{+}IgD^{+},CD27^{-}IgD^{-} and CD27^{-}IgD^{+} cells for sorting. B. Analysis of cytokine production by sorted cells.

Supplemental Figure 4

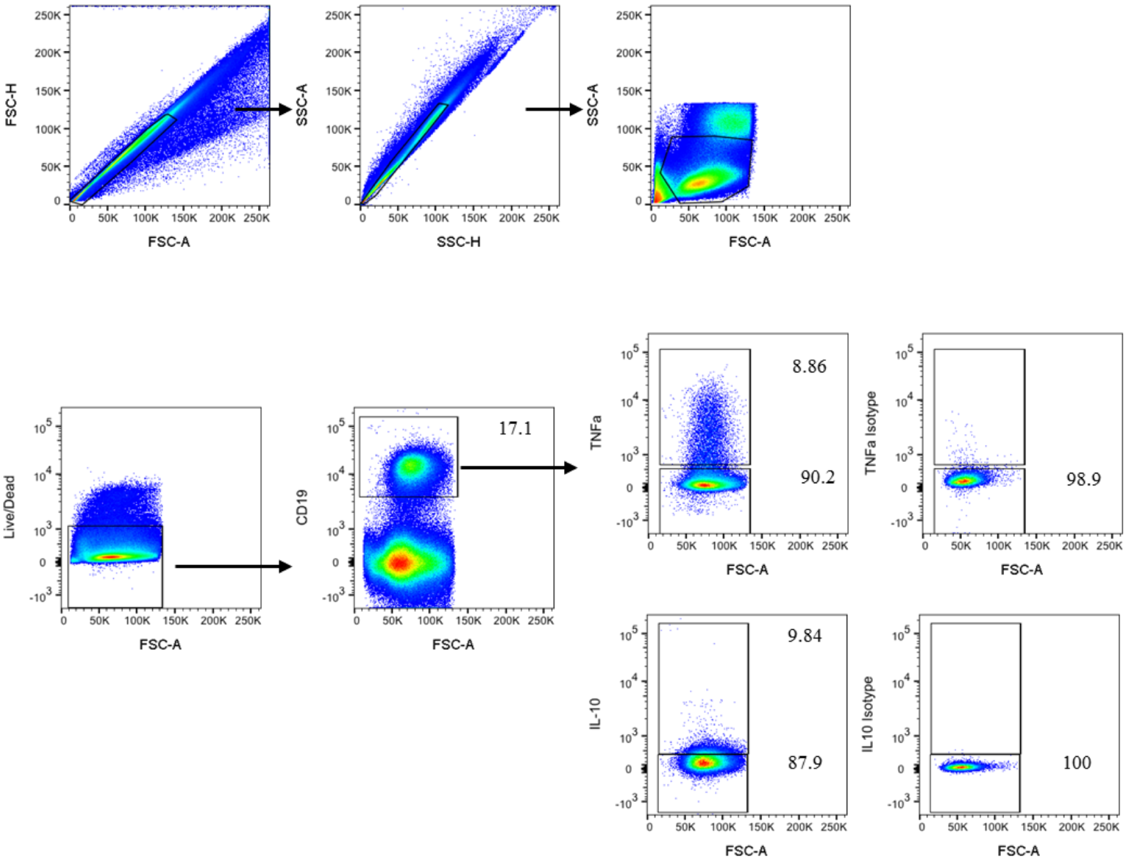

Supplemental Figure 4. Gating strategy for analysis of TNFα and IL-10 production by total CD19$^+$ populations

Supplemental Figure 5

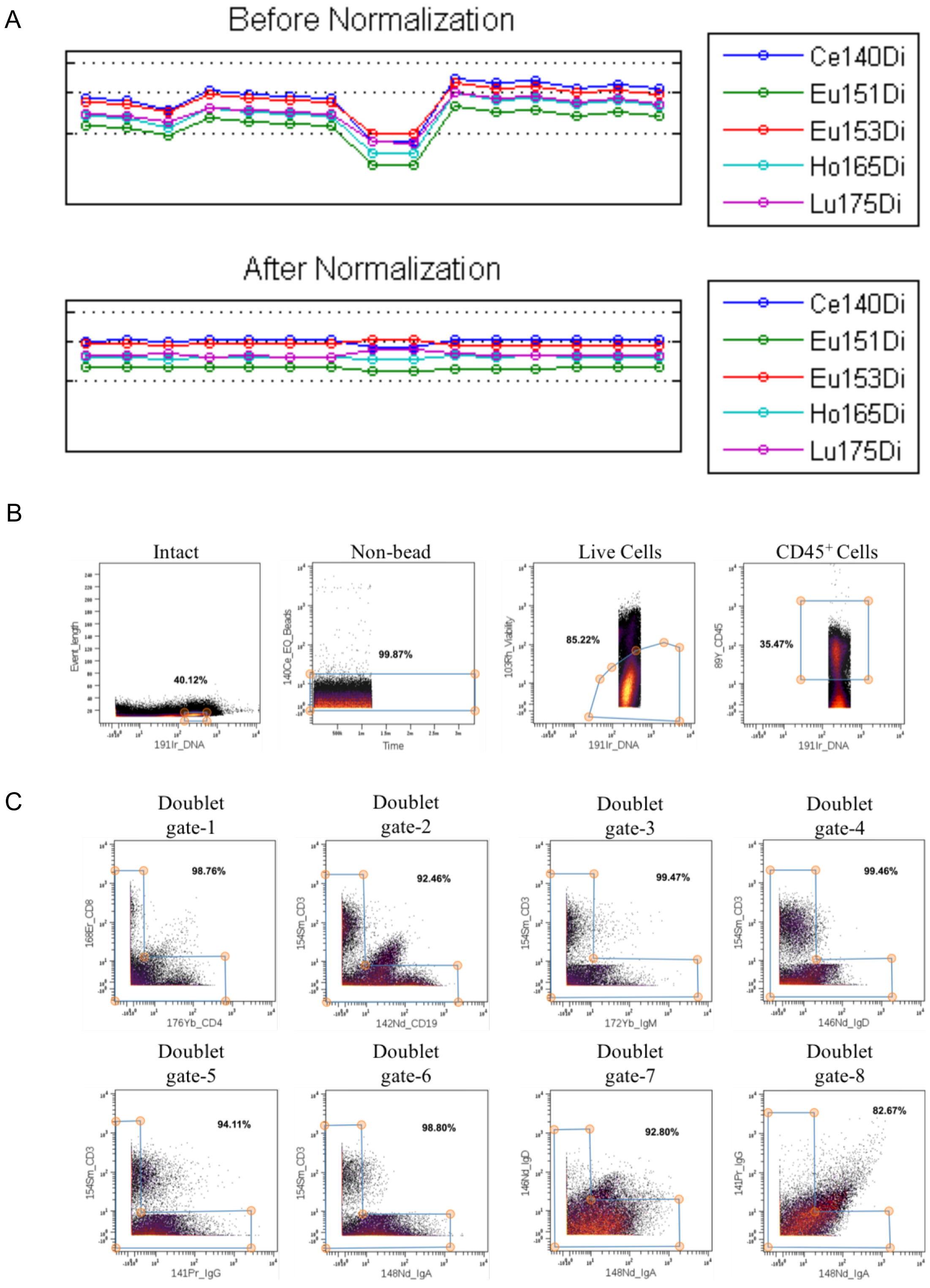

Supplemental Figure 5. Quality control and preliminary gating for mass cytometry. A. Bead normalization. B. Preliminary gating for analysis of $CD45^{+}$ cells isolated from GALT. A gate was created around intact cells that ensured that all relevant events were captured. C. Subsequent gates cells excluded doublets and the remaining minority of cells with implausible marker combinations.

Supplemental Figure 6

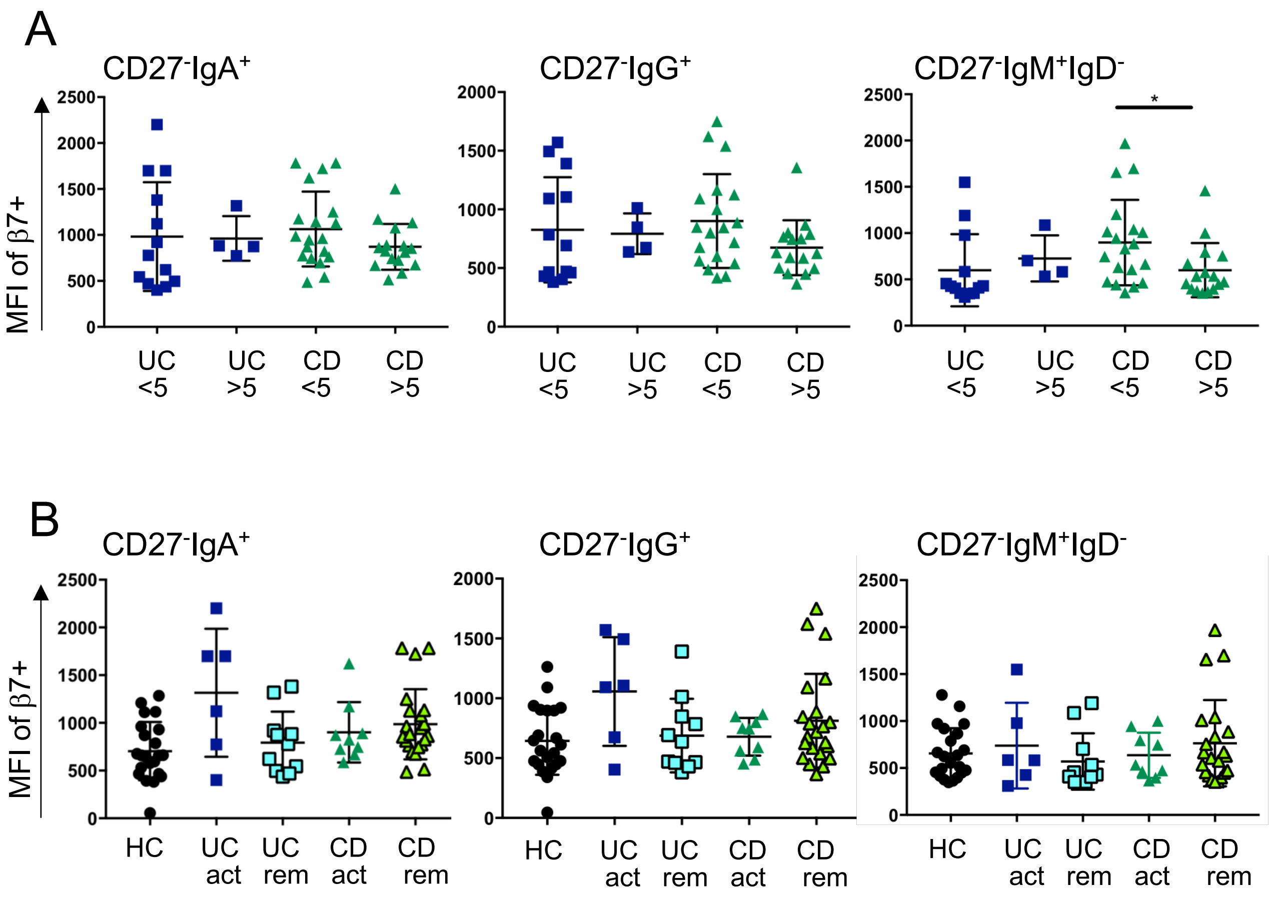

Supplemental Figure 6. MFI of β7 integrin expression by blood CD27-IgD- subsets in CD and UC patients stratified according to CRP levels and remission status. The MFI of β7 expression by three subsets of CD27-IgD- B cells (CD27-IgA+, CD27-IgG+ and CD27-IgM+). For A, the values of <5 and >5 refer to CRP levels. UC<5 n=13, UC>5 n=4, CD<5 n=19, CD>5 n=16; for B, ‘act’ refs to active disease and ‘rem’ to remission. HC n=22, UC active n=6, UC remission n=11, CD active n=9 and CD remission n=22. Data is analysed by Mann-Whitney U test where $p<0.05$=*.
